# From Mary Shelley to Netflix: a Pan-European perspective on public communication of neuroscience and neurotechnology

**DOI:** 10.3389/fnins.2024.1278640

**Published:** 2026-02-20

**Authors:** Ángeles Consuelo Gallar Martínez, Alicia De Lara González

**Affiliations:** ^1^Unidad de Cultura Científica y de la Innovación, Miguel Hernández University, Elche, Spain; ^2^GICOV Research Group, Human and Social Sciences Department, Miguel Hernández University, Elche, Spain

**Keywords:** science communication, neuroscience communication, scientific culture, cultural impact of science, neuroscience in film, neuroscience in art, neuroscience in children’s literature, history of neuroscience

## Abstract

Scientific knowledge of the human brain has captivated the public’s attention and sparked their imagination for centuries. Comprehending the inner workings of the mind and the underlying molecular and physiological aspects of the central nervous system has long been the defining theme of contemporary Western scientific culture. Even as the focus has arguably shifted towards genomics in the early 21st century, the brain continues to hold the spotlight in science communication, perhaps bolstered by the hype surrounding Artificial Intelligence. Neuroscience and neurotechnology, with their connections to culture, identity, economic progress, and health, remain subjects of fascination for people of all ages who seek to understand the present and future implications of research in these fields. In this work, we explore 10 distinct ways of communication dealing with the subject of the brain, the mind, applied neurotechnology, and what makes us, and possibly other things, human. We examine European literature, material culture, and various film formats to gain insights into these captivating subjects. Instances like Mary Shelley’s “Frankenstein” exemplify the historical fear of science. At the same time, TED Talks and documentaries have emerged as influential platforms for scientific communication. The intersection between art and brain imaging helps visualise abstract concepts. The gamification of thought experiments is an accessible tool for the public to understand complex cognitive phenomena. And, despite a lack of accuracy, science fiction can spark public debates on ethical issues involving the conscience of robots or the privacy of our brain data.

## 1 Introduction

As neuroscience advances, there is growing pressure within the scientific community to engage in discussions about research findings and the ethical implications of their work (Illes et al., 2010, p. 61). Society has a vested interest in comprehending the intricacies of the brain, and this interest can be traced back not only to recent times but arguably to the past couple of centuries. During the 17th century, there was a notable shift in the understanding of the self and the mind, moving away from the Aristotelian framework that previously dominated Western thought. The concept of the soul as the driving force behind organic functions gave way to recognising the brain as the seat of mental faculties (Vidal, 2009, p. 15). Consequently, this shift in academic thinking necessitated a surge of cultural expressions that reflected this new paradigm. The mind was no longer attributed to divine powers; instead, it became a distinctly human attribute encompassing thoughts, memories, emotions, desires, and more. The brain emerged as the domain of this newfound knowledge.

“The task of modern neuroscience is as simple as it is formidable,” wrote neurobiologist Eric Kandel when summarising the origins of modern neuroscience: to offer comprehensive explanations in cellular and molecular terms for various aspects of normal mental processes, including perception, motor coordination, feeling, thought, and memory. Additionally, neuroscientists aim to understand and explain the functional disorders arising from neurological and psychiatric diseases (Kandel, 1982, p. 1).

Emerging as a distinct field in the second half of the 20th century, neuroscience is a relatively new scientific discipline, despite its historical roots dating back to ancient Egypt, when the term ‘brain’ was first recorded, around 1700 b.c.e. Later, about 300 b.c.e., Aristotle made a distinction between the *‘enkephalon’* (cerebrum) and ‘*parenkephalis’* (cerebellum; Brown, 2019, p. 4, 7). This is to say that humans have been talking about the brain for thousands of years, whether in the context of science, religion, or notions of personhood. Nowadays, terms such as ‘nerves’ and ‘brain function’ have transcended the confines of scientific discourse and have become widely used in various fields of study and everyday language.

### 1.1 Neuroscience as a topic of scientific culture

Nonetheless, speaking can be different from communicating. Communicating neuroscience implies the dissemination of accurate accounts of research to the public and engagement activities in two-way forums for dialogue (Kappel and Holmen, 2019, p. 2). The current approach to this undertaking is driven by the overarching goal of democratising neuroscience literacy. Also, it aims to open discussion on what the brain can tell us about ourselves to inform public policy and individual lifestyles. Moreover, it is widely recognised that fostering a robust scientific culture among children is instrumental in guiding them towards STEM careers and facilitating the development of a democratic and sustainable society for future generations (Schusler and Krasny, 2014, p. 367; Ahmed and Mudrey, 2019, p. 202). These tasks are undertaken by the scientific community working jointly or in parallel with neuroscience communication specialists, as well as mass media outlets, social media channels, and commercial third parties who may or may not be specialised in the field.

Scientists who communicate often do so because they believe it is the right thing to do or in recognition of the public’s right to knowledge (Illes et al., 2010, p. 67). However, not all of them get deeply involved in science communication. The substantial challenges facing neuroscience communication involve this discipline being prone to misinformation, inaccurate reporting (van Atteveldt et al., 2014, p. 2), and the prevalence of ‘neuromyths’ (Betts et al., 2019, p. 8). For instance, animal research is a considerable barrier (Miguel-Batuecas et al., 2023, p. 12). Moreover, the complexity of the central nervous system makes it extremely challenging to convey accurate and understandable information to non-experts. Determining what constitutes an “appropriate simplification” of scientific terms or identifying when a topic has been oversimplified or inadequately translated for the public is an ongoing uncertainty (Johnson and Littlefield, 2011, p. 283; Dempster et al., 2022, p. 17).

Still, researchers are encouraged to communicate their results as a requirement of public funding. As a deterministic result, expertly summed up by Arboledas-Lérida, this can pose an additional ‘capitalist pressure’ upon individual researchers and universities and has -in fact- spurred a ‘cultural shift’ among academics who now regard science communication as a primary component of their activity (Arboledas-Lérida, 2023, p. 202). This phenomenon was exacerbated by the global crisis during the COVID-19 pandemic -and infodemic (Colombo, 2022), with added societal and media pressures to communicate complex scientific concepts and recent scientific results, even before the peer-review process had concluded (Fraser et al., 2021, p. 2; van Schalkwyk and Dudek, 2022, p. 608).

In the meantime, opportunities for Neuroscience communication are on the rise. Public engagement initiatives, such as The Brain Awareness Week by the Dana Foundation, are promoted to foster public enthusiasm and harvest support for brain science and encourage youth to pursue a scientific career (McNerney et al., 2009, p. A61). This initiative, as well as scientific communication in general, has branched out to social media. Research institutes, such as the Institute for Neuroscience in Alicante (Spain), and scientific societies, such as the Society for Neuroscience or the Kavli Foundation, maintain social media accounts. More research is required to evaluate if the activities of these types of institutional accounts are reaching the general public or, instead, reinforcing connections among the scientific community. The same question can be posed about individual researchers who are active on social media. In Spain, only some active researchers (i.e., still conducting scientific research in a laboratory) have successful social media accounts that reach beyond their peers. A recent study by Buitrago & Torres-Ortiz found that YouTube channels belonging to scientific institutions have a far smaller impact and engagement than science influencer channels (Buitrago and Torres-Ortiz, 2022, p. 140).

Additionally, the nature of science, which constantly questions and challenges beliefs, adds to the difficulty of continuously educating the public at a pace and scale that may be overwhelming, even for those working in scientific fields. As we will see further in the discussion, the philosophical and religious saliences to mind and body and the personal and societal impact of neurological diseases and neuro diversities must also be considered. Despite these challenges, engaging in science communication allows neuroscientists to understand better how their work aligns with the broader academic framework and the social context (Illes et al., 2010, p. 66).

Nevertheless, sometimes, other actors not involved in research speak for neuroscience. Whether in movies, books, figurative art, or TV shows, neuroscience and neurotechnology appear as a subject of narrative, character, and cultural entity. One interesting question is how these outlets shape the ideology of brainhood we currently live on, in which humanities and social sciences have been transformed to accommodate the knowledge of the human brain (Vidal, 2009, p. 22).

## 2 Methods and limitations

As important as it is to consider science history, culture, and communication from a global perspective and avoid Eurocentric biases, this paper focuses on European instances of neuroscience communication and scientific culture within the context of The NeurotechEU project. This Pan-European initiative represents the collaborative efforts of the scientific community in addressing societal challenges, particularly related to the ageing population, and educating the next generation of neuroscience researchers and neuroengineers. To provide a diverse perspective, we have compiled various communicative product examples from countries that are or have been NeurotechEU members: France, Germany, Hungary, Iceland, the Netherlands, Romania, Spain, Sweden, Türkiye, and the United Kingdom.

In choosing the examples, three domains of scientific culture outlets were considered: Writing, Arts and Spaces, Video Narratives and Social Media. These contain several subgenres, such as fiction novels, movies or art installations, which are prone to travel across language barriers further than other forms of scientific communication, such as journalism. After a thorough search, and matching these subgenres with several neuroscience-related keywords, the following examples were chosen:

**Table 1:** 

**Writing**		
**Fiction novel**		
*Frankenstein*, or *The Modern Prometheus*, Mary G. W. Shelley (1818)	United Kingdom	First evidence of the social perception of science in a mass distributed cultural product. According to Cambra-Badii et al. (2021, p. 1), Frankenstein can be useful for analysing bioethical issues related to advances such as artificial intelligence, genetic engineering, and cloning.
**Autobiographies**		
*Memorias de mi vida* (*Recollections of My Life*), Santiago Ramón y Cajal (1901)	Spain	Even though scientists’ autobiographies are problematic as a historical source (Söderqvist, 2002, p. 42), they have largely contributed to the public perception of scientists.
**Children’s literature**		
*Bräkiga bokstäver* (*Noisy Letters*), (Helena Bross & Mati Lepp, 2018)	Sweden	Example of cultural outlet about neurodiversity in childhood. Neuroscience has contributed to the understanding of the biological basis of reading disability. Treatment requires a social, broader approach (Eden and Moats, 2002, p. 1083).
**Arts and Spaces**		
**Museum exhibition**		
Humania Exhibit, NEMO (2019)	The Netherlands	Representative of the dominant model of science museum today, which has gone from showing to demonstrating through experimentation (Schiele, 2008, p. 33).
Art Installation		
*Connectome Architecture* (Refik Anadol, 2021)	Türkiye	As digital art and culture change the creative process and the way of construction meaning (Lovejoy et al., 2011, p. 326), this installation exemplifies how data science and brain imaging have permeated aesthetics and culture.
Video narratives and social media		
Science-fiction films		
*Metropolis* (Fritz Lang, 1927)	Germany	An early example of narratives against the dehumanisation of modern life utilising references to artificial intelligence, in conflict with the aspiration to build humanoid robots (Siciliano and Khatib, 2019, p. 15).
YouTube		
Reykjavik TEDx Talks (2014)	Iceland	A mainstream social media format which has transformed the way scientific knowledge reaches the public, no longer requiring the participation of researchers (Sugimoto et al., 2013, p. 668).
Short film		
*Erica: Man Made* (Ilinca Caligareanu, 2017)	Romania	An example of editorialisation in the communication of neurotechnology. Scientific journalism can fall victim to the ‘dazzle effect’ of neurotechnology and completely avoid questioning the potential dangers of brain emulations, such as self-improvement (Sandberg, 2014, p. 453).
Serial show, science-fiction		
*Osmosis* (Audrey Fouché, 2019)	France	Representative of how science fiction mixes and confuses real and made-up scientific concepts (Petersen et al., 2005, p. 350).
Documentaries		
*Alzheimer* (Istvan, 2020) and *Hüség* (Szekeres Csaba, 2022)	Hungary	Exemplifies the role of scientific communication in achieving social goals (Chattoo, 2020, p. 21).

The selected examples serve as a foundation for in-depth discussions on neuroscience communication in society and warrant further scholarly exploration in scientific literacy, scientific culture, and science communication. The period reflects the long public conversation around neuroscience and neurotechnology in Europe.

## 3 Discussion

### 3.1 Fiction novel: Frankenstein or The Modern Prometheus, Mary G. W. Shelley (1818), United Kingdom

It has been argued that scientists and scholars interested in neuroethics offer a compelling starting point for advancing communication in neuroscience (Illes et al., 2010, p. 67). And so does this research paper, starting with the not too technical but quite philosophical approach to the core of neuroscientific research, Mary Godwin Wollstonecraft Shelley’s Frankenstein or The Modern Prometheus, first published in 1818.

In the novel, a young Victor Frankenstein becomes consumed with the idea of creating life during his time at university. He assembles a creature from various body parts and animates it. However, upon seeing the Creature’s appearance, Victor is horrified and rejects him. The Creature abandoned and rejected by society, experiences profound loneliness and seeks his creator, demanding a companion. Victor reluctantly agrees and begins creating a mate for the Creature. However, he ultimately destroys the unfinished creations out of fear that they will reproduce. In retaliation, the Creature kills Victor’s wife and, eventually, Victor himself.

A self-educated 19-year-old, Mary Shelley wrote this story on a dare. Despite her tender age, she penned it within a profoundly dramatic context. Tragedy struck early in her life when her mother passed away shortly after her birth. Her father, in turn, remarried and disregarded her. Mary’s lover, the poet Percy Shelley, with whom she eloped, led a complex life. While he associated with well-regarded philosophers of science, he mingled with morally questionable figures like Lord Byron and engaged in extramarital affairs. Furthermore, Mary Shelley endured the heart-wrenching loss of all but one of her children, leaving a lasting impact on her views of death.

It is crucial to note that this context does not serve to portray Mary as a mere victim of circumstance but rather as a person deeply engaged in contemplations of motherhood, mortality, scientific inquiry, and moral obligations towards others. Some scholars argue that the actions of Victor, the protagonist in Frankenstein, symbolise the pursuit of alchemy (e.g., Smith, 1994, as cited in Ginn, 2013, p. 175), while alternative interpretations view him as a representative of the intellectual climate of the time—an embodiment of the natural philosopher (e.g., Vasbinder, 1984; Reichardt, 1994; Ketterer, 1997, as cited in Ginn, 2013, p. 175). Additionally, this character offers a stark juxtaposition between two types of scientists prevalent during that era: the pensive philosopher exemplified by Erasmus Darwin and the ambitious conqueror of nature embodied by Humphry Davy (Ginn, 2013, p. 176). Delving into Frankenstein, readers embark on a thought-provoking journey that invites reflection and moral deliberation on the authority of science over the natural world, what psychologist John Dewey describes as a “moral rumination” (Martinez-Conde et al., 2019, p. 8286).

How has this book shaped the popular perception of neuroscience worldwide? Even if one does not read the novel, the numerous adaptations in theatre, television, and cinema have ingrained in popular culture the idea that the brain defines a person, separate from their physical body (El Vidal, 2011, p. 4). Popular ideas about the brain are vital since they can influence neuroscientists’ direction in their research (Brown, 2019, p. 13).

**Figure 1:**
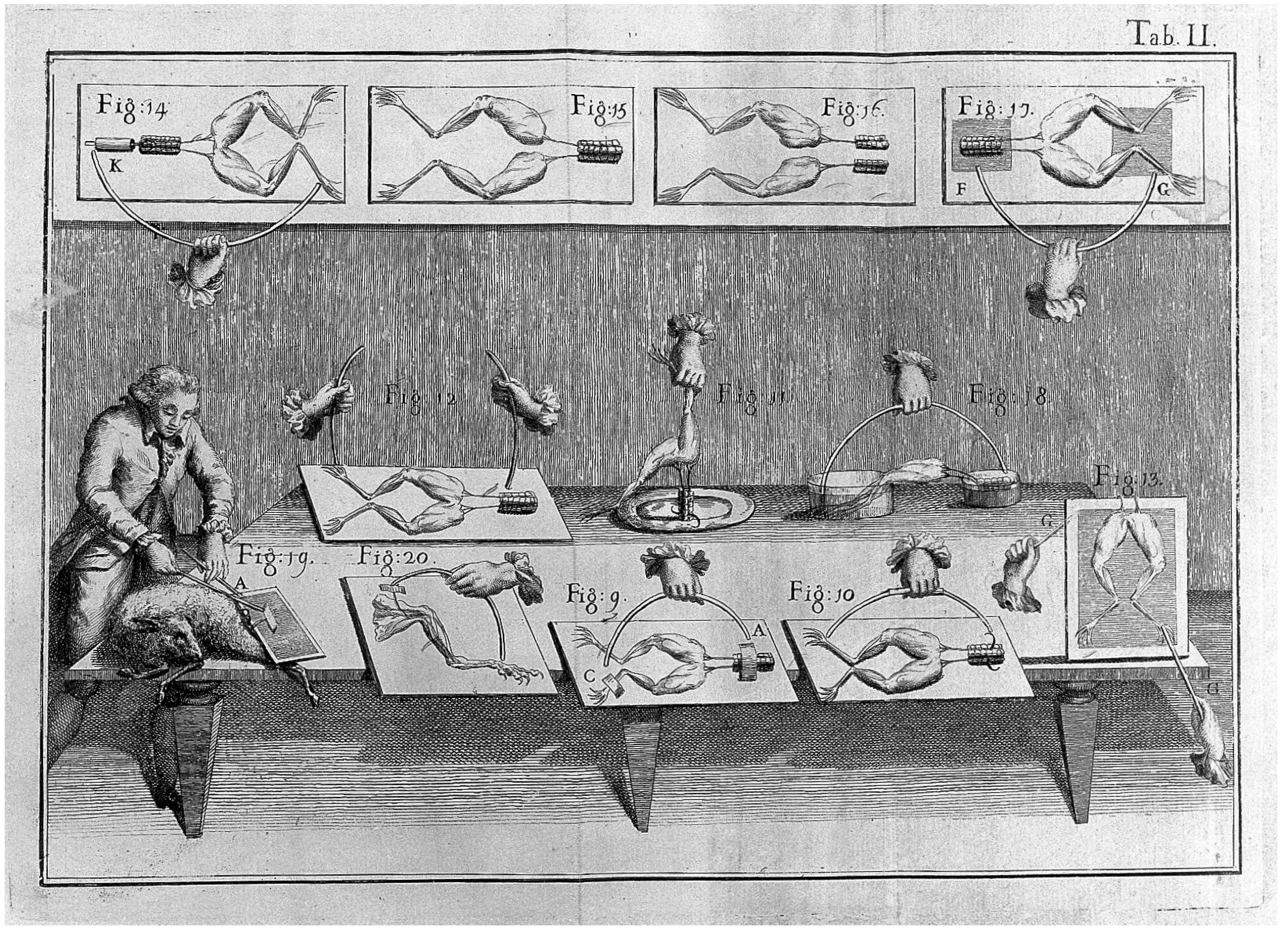
*De viribus electricitatis in motu musculari commentarius. Cum Joannis Aldini dissertatione et notis*. Luigi Galvani, 1792. Held by the British Library. Shelfmark: 1502/250. Public Domain.

Shelley chose not to explain how the Creature is animated in the novel’s first edition. Still, she added a lot of scientific detail in the second edition of 1831. Just like current developments in neuroscience and neurotechnology raise high societal expectations, Luigi Galvani’s early experiments in bioelectricity became a sensation during that period (See Figure 1). Galvani proposed that electricity flowed through nerves, challenging the prevailing belief in the existence of an animal spirit. These experiments, along with others conducted then, led to the popular notion that electricity could restore life to a dead body.

Interestingly, even the Shelley family turned to this technique when Percy and Mary’s second child, William, succumbed to malaria: ‘he was once reanimated after the process of death had commenced, and he lived four days after’, wrote the poet in a letter (Ruston, 2014). The success of electric reanimation was limited, at best. As a result, Mary Shelley’s work has become the first science fiction novel and arguably the first cultural symbol of the promise and disappointment cycle in societal expectations of biotechnology, as Brown and Michael (2003) described.

The novel also delves into the nature versus nurture debate. Mary Shelley positions herself on behaviourism: the creature can feel and express love and empathy. It is the causal factors, such as being rejected by society and being abandoned by her creator, that cause its homicidal behaviour. In contrast to this premise, the author was inspired by the concept of cortical localisation of functions by Gall and Spurzheim, whose works she had read shortly before conceiving the story (Ginn, 2013, p. 180). The observations of Joseph Gall and his disciple Johan Spurzheim gave rise to the pseudoscience of phrenology - then known as physiognomy - which stated that certain mental functions are located in specific parts of the cerebral cortex. Through neuroanatomical studies, they tried to demonstrate that people’s cognitive ability, mental health, and personality can be extrapolated from their physiognomy. While Shelley did not explicitly attribute the Creature’s personality to the shape of its head, she did borrow descriptive techniques from phrenology to describe several characters in the novel. For example, Elizabeth, Victor’s fiancée, was of a sensitive and sweet nature, as indicated by the breadth of her eyebrows, clear blue eyes, lips, and the profile of her face.

On the other hand, Victor Frankenstein represents the evolving nature of science itself, transitioning from uneducated notions and mystical beliefs to empirical scientific methods (Ginn, 2013, p. 185). This transformation mirrors the changes occurring in the scientific landscape during Mary Shelley’s time. This aspect of the book holds particular significance for science historians, as it offers a unique perspective on the changing understanding of science. Although novels are not typically considered primary sources for historical research, other forms of literature, such as autobiographies, often serve as valuable resources, which we will explore further in our discussion. For Passmore (1983, p. 25), the novel is an early example of fear of science.

### 3.2 Autobiographies: *Memorias de mi vida (Recollections of My life)*, Santiago Ramón y Cajal (1901), Spain

Santiago Ramón y Cajal (1852–1934) set the foundations of modern neuroscience. His theory elevated the neuron to the status of the fundamental unit of the nervous system, encompassing its anatomical, physiological, genetic, and metabolic aspects. Cajal’s groundbreaking studies played a pivotal role in fostering an environment conducive to the development of modern neuroscience, surpassing the contributions of any other researcher of his time (DeFelipe, 2002, p. 483).

In addition to his groundbreaking research, Cajal was an incredibly prolific writer. According to one account by Vázquez-Tapioles (2005) (as cited in Collado-Vázquez and Carrillo, 2016, p. 471), Cajal’s body of work includes an impressive catalogue of 391 publications. Within this extensive collection, he authored several books intended for a non-scientific audience, along with science fiction short stories. These writings demonstrate Cajal’s commitment to reaching a broader readership and his ability to communicate complex scientific concepts in accessible ways.

Cajal’s autobiography has been an object of study in and of itself. Published in 1901 under the title *Recuerdos de mi vida*, it stands as an early example of ‘childhood and youth stories’, a subgenre of autobiographical literature that emerged in Spain during the early 20th century (Fernández, 2006, p. 71). Cajal’s recollections exhibit a unique prose style found in contemporary fiction novels that offers a heightened sense of perception and delves deeper into the character’s inner self, surpassing the limitations of traditional historicist autobiographies. Fernández argues that Cajal wrote his memoirs with a specific agenda: actively participating in Spain’s cultural and societal “regeneration” (p. 72).

A self-proclaimed patriot, the Nobel laureate (p. 74) took it upon himself to revitalise Spanish society following the loss of the empire’s last overseas colonies. His intention would account for his prose’s ‘messianic’ tone, as Fernández puts it (p. 73). According to this author, Cajal portrays himself as sacrificing his artistic aspirations during his youth in favour of a new sense of “scientific patriotism.” Cajal, both as the author and the character in his memoirs, embodies the archetype of the oedipal hero, representing the ideal of a patriot-scientist. Consequently, the first volume of his memoirs can be seen as a psychological exploration, where a father-son conflict shapes the celebrated neuroscientist.

Studying his autobiography, del Barrio Gándara (2017) has retrospectively found that Ramón y Cajal suffered bullying from his peers and some adults. She points out that by reading the scientists’ memoirs, one can have deep insights into the causes of abuse and how it affects the victim.

“Yo opuse al principio algunas resistencias a los juegos brutales [.], pero el espíritu de imitación pudo más que los sabios consejos de mis padres [..] Algo hubo, con todo eso, en que mi caballerosidad nativa no transigió jamás: fue el abuso de la fuerza con el débil, así como la agresión injusta y cruel. [..] Merecida o exagerada, mi fama de pícaro y de travieso crecía de día en día. [..] Y sin embargo, y a pesar de todo, yo era un infeliz.” (Cervantes, 1997).

[‘I resisted those beastly games at first, [..] but the spirit of imitation overpowered the wise advice from my parents. [..] There was something in which my native chivalry never compromised: the abuse of force over the weak and the unfair and cruel aggression. [..] Deserving or not, my reputation as a scoundrel and a scallywag grew by the day [..] And yet, and despite everything, I was just miserable.’]

Naughty Cajal was just a kid learning to defend himself. He mastered the sling and used the forest as an impromptu ‘CrossFit’ gymnasium to muscle up, scare away his aggressors, and fit in a violent teenage-angst-ridden surrounding. Interestingly, this aspect of his personality has been prominently featured in various adaptations of his autobiography aimed at younger audiences, such as *De travieso a sabio* [*From Puckish to Wiseman*] (María Dolores de Ygartua Landecho, 1957) and *Cajal el travieso* [*Cajal the Menace*] (Esteban Rodríguez Serrano, 2010; works referenced in Collado-Vázquez and Carrillo, 2016, p. 473).

According to Anaya-Reig and Romo (2017), some of Cajal’s books, including his memoires and certain non-specialised works, contain valuable psychological insights into scientists. These authors argue that contemporary knowledge in the field of Psychology of Science has confirmed many of Cajal’s observations regarding the psychosocial aspects of scientists, scientific reasoning, and creativity. As such, Cajal’s memoirs contain discussions of his scientific work on nerve degeneration and inspire subsequent generations of neuroscientists (Trumpler, 1994, p. 543).

Nevertheless, as his memoirs were translated and published in different historical contexts, the perception of Cajal’s legacy within the scientific community varied. During the Spanish Civil War, George Sarton emphasised the importance of discussing Cajal as one of the great men of Spain, given the country’s present circumstances. However, Sarton’s interpretation of Cajal’s autobiography has an even greater after-the-fact approach. It is worth noting that some of the justifications for Cajal’s achievements in that era would be considered politically incorrect by today’s standards, as they emphasised racial qualities and the strength of his heritage (Sarton, 1938, pp. 118–122).

In neuroscience communication, Cajal’s memoirs serve as a multifaceted narrative of his personality and function as a tool for propaganda, constructing the image of an unattainable genius, a saint-like figure. Within his memoirs, the histologist reflects on the transformation of the scientific profession during his lifetime, transitioning from an individualistic and entrepreneurial pursuit to a state-funded job position. While the prevailing interpretation of his autobiography portrays a story of unwavering determination and sheer willpower, Fernández highlights that beneath this facade lies a sense of nostalgia for the artistic vocation that a young Santiago had abandoned (p. 88).

Just as a celebrated scientist, Cajal could have been a keen photographer. An abusive teacher often sent Santiago to a dark room. In this confined space, he made a remarkable observation - as light passed through a crack in the wall, it projected an inverted image of the outside, leading him to discover the properties of the camera obscura. According to Bentivoglio (1998, p. 1), Cajal’s passion for drawing and his appreciation for visual aesthetics significantly shaped his future scientific endeavours. Similar to Andy Warhol’s famous Campbell Soup series, Cajal’s drawings of cells in the nervous system have become iconic, finding their place in numerous neuroscience research laboratories. At the Institute of Neuroscience in San Juan de Alicante, Spain, visitors are greeted by wall sculptures based on Cajal’s drawings of Purkinje cells with their distinct dendritic structures upon entering the east wing. A large metallic reproduction of “The retina according to Cajal” adorns the west entrance. These sculptures, created by ophthalmologist José Belmonte González in the 1980s, pay homage to Cajal’s influential illustrations.

Cajal felt science was a calling for progress. He believed his efforts would permeate the whole of Spanish society, and beyond. Following Cajal’s era, neuroscientists have increasingly been tasked with addressing societal challenges. Taking inspiration from the United States, the Commission of the European Communities proclaimed the ‘European decade of brain research’ (Schleim, 2014, p. 1), a strategic initiative supported by economic funding that has continued to this day. Presently, initiatives like The Neurotech EU have taken it upon themselves to contribute to pressing social issues, such as prevalent brain diseases associated with an ageing population and the imperative of supporting the next generation of neuroscience researchers who will confront these challenges. While there are voices expressing scepticism about this approach and questioning whether we might be overestimating the impact of neuroscience at the expense of other drivers of social and cultural change, media representations of neuroscience tend to be biased towards a favourable portrayal of certain aspects (Schleim, 2014, p. 2).

Positive representations of scientific results are a common issue in the field (van Atteveldt et al., 2014, p. 9). One contributing factor is the publication bias favouring the dissemination of positive findings over negative ones (Gallar and Gallar, 2021, p. 162). Press releases from universities and research institutions often highlight partial results as groundbreaking discoveries. As professionals in science journalism and institutional science communication, we acknowledge that such exaggerations exist but believe they are not the norm. However, media outlets tend to replicate and amplify these occasional misinterpretations, resulting in inaccurate reporting of scientific results. This problem is particularly evident in traditional news programmes, which have limited time for reporting and brief segments for each piece. In contrast, formats like documentaries, with longer production periods and more narrative flexibility, can serve as better vehicles for science communication, even though the perspectives of directors and producers still influence the narratives they present.

### 3.3 Documentaries: Alzheimer (2020), by István Kollár, and Hüség (2022), by Szekeres Csaba, Hungary

“I’m trying to explain something behind which there is a whole life. It is impossible to explain.” These poignant words, spoken through tears by the life partner of a patient who has advanced Alzheimer’s disease, encapsulate the challenge of not only conveying the scientific aspects of central nervous system pathologies but also capturing the lived experience of those affected by brain diseases. In his, 2020 documentary titled *Alzheimer*, Hungarian filmmaker István Kollár delves into the struggles faced by individuals with Alzheimer’s, their families, and the medical professionals and researchers working in this field. The film presents both the hardships encountered and some encouraging examples of social initiatives aimed at supporting Alzheimer’s patients and their loved ones. It emphasises the urgent need for societal changes and increased support from government institutions, given the profound impact on caregivers and the slow progress in finding a cure for the disease.

Equally heart-wrenching is another Hungarian documentary *Hüség* (*Loyalty*), directed by Szekeres Csaba, which premiered 2 years later. The film follows the last days of Anna and Antal, a couple married for 45 years. Presented in a unique format reminiscent of a home movie, the documentary depicts the gradual deterioration of their relationship as Anna’s behaviour becomes increasingly erratic, and she is eventually diagnosed with Alzheimer’s disease. We witness the heartbreak as she reaches a point where she no longer recognises Antal. In contrast, Kollár’s film *Alzheimer* takes a different approach, exploring the scientific and societal challenges surrounding the disease and individual struggles.

Yet, both documentaries deeply engage viewers on intellectual and emotional levels, tapping into the power of storytelling. When information resonates with audiences on a personal level, it generates interest and builds confidence. Although people tend to seek confirmation of their biases and beliefs in their choice of information sources (Charness et al., 2021, p. 20), storytelling can challenge this pattern. Storytelling can mimic personal contact, creating empathy and serving as positive reinforcement (Burke et al., 2015). Moreover, narratives offer intrinsic benefits by enhancing information processing and facilitating long-term memory retention (Dahlstrom, 2014).

But what does storytelling mean in the context of neuroscience communication? A narrative can be a description of events that serves to illustrate a set of aims or values (Oxford Learners Dictionary, 2023). In scientific storytelling, the narrator employs voice, character development, suspense, and vivid descriptions to connect various story elements and deliver a compelling message. According to Martinez-Conde et al. (2019, pp. 8285, 8286), narrative storytelling can captivate lay audiences and generate enthusiasm for significant scientific discoveries that may not have immediate practical applications. They also note that evoking emotions and a sense of wonder can sometimes be achieved by deviating from a straightforward narrative structure.

### 3.4 Art: *Connectome architecture* (Refik Anadol, 2021), Türkiye

Not telling a straightforward story is sometimes the very point of contemporary art. Indeed, art often challenges traditional storytelling conventions by embracing explicit or implicit narratives. One artist who explores new forms of storytelling in the age of big data is Refik Anadol, born in 1985 in Istanbul, Türkiye. According to an article on KCET.org (2016), Anadol believes that the vast amount of information available in the era of big data can give rise to a novel kind of storytelling.

In neurotechnology, big data is crucial in aggregating information from diverse experiments to uncover patterns related to brain activity. Through large-scale analyses, researchers aim to identify robust relationships between brain function, cognitive processes, and task performance. By combining heterogeneous datasets, these mega-analyses seek to reveal universal features that connect neural activity, cognitive states, and behaviour (Bigdely-Shamlo et al., 2020).

At the same time, imaging technology has become a significant area of scientific research focusing on understanding higher brain functions such as emotions. Electroencephalography (EEG) provides valuable temporal information, while Functional Magnetic Resonance Imaging (fMRI) offers high spatial resolution (Grima-Murcia, 2017, p. 20). Neuroimaging, including machine-learning approaches, has the potential to shed light on brain-behaviour relationships (Raz, 2011, 270).

An intersection between art and neurotechnology highlights the potential for innovative storytelling approaches that leverage big data and brain imaging to explore the complexities of the human mind and its relationship with the external world.

Refik Anadol is renowned for his pioneering work in the aesthetics of machine intelligence. His exhibition, *Space* in Istanbul, is the city’s most visited exhibition (refikanadol.com, 2023). Anadol, based in Los Angeles, California, explores data narratives and the impact of ubiquitous computing on humanity in the age of Artificial Intelligence. He employs these tools to transform digital data into physical designs such as “data sculptures and paintings,” immersive installations, and performances. Through his work, Anadol invites the audience to experience alternative realities and contemplate what it means to be human in the context of emerging technologies.

In the art installation *Connectome Architecture* exhibited at Sense of Space, La Biennale di Venezia in 2021, Refik Anadol collaborated with Turkish-American neuroscientist Gökhan S. Hotamisligil to create a 3D printed sculpture that represented the visualisation of data points related to learning, memory, and emotions in the human brain. Hotamisligil collected a dataset of 4,500 individuals of different age groups, and a machine-learning algorithm processed the MRI scans to generate imagined neural networks. This artwork gave the audience a visual representation of the intricate neural connections contributing to human experience.

Another notable work by Anadol is *Latent Being* (2019, Kraftwerk, Berlin, Germany), where visitors interacted with the artwork using biofeedback technology, providing real-time data that fueled an artificial thinking process. This exploration aimed to capture and visualise the intangible human experience of cities. Given the complexity of this social and cognitive phenomenon, Anadol’s studio embraces a multidisciplinary approach, involving not only designers and architects but also data scientists and researchers from various fields.

Anadol’s use of neuroimages and data visualisation techniques can be seen as part of the ‘neurorealism’ trend, which refers to the persuasive power and credibility of brain images in shaping public perception of neuroscience (Racine et al., 2005; Popescu et al., 2016; Casini, 2017; Gruber, 2017). His work highlights the influence of advances in cognitive science and sensing technologies, which have paved the way for a data-driven understanding of various aspects of life, including our surroundings and cultural approaches to aesthetics (see other examples of MindSpaces provided in Alvanitopoulos et al., 2019). Additionally, Anadol uses the captivating allure of neuroimages, employing what has been described as the ‘dazzle effect’. Neurorealism, as a concept, significantly contributes to the credibility and persuasive power of brain images (Robillard and Illes, 2011, p. 118).

On the other hand, images can give the illusion of objectivity and are prone to misinterpretation. According to Soares et al. (2016), neuroscientists must exercise caution and precision when interpreting and representing fMRI results. The allure of neuroimaging can be seductive, leading to the danger of oversimplification and the tendency to draw unwarranted conclusions through reverse inference. This critique often applies to certain forms of science communication, particularly within neuroscience and neurotechnology.

Anadol expresses his perspective: “Instead of trying to make humans more machine-like, I find it more interesting to make machines more human” (Sleek-mag.com, 2020). This sentiment is a recurring theme within the cultural discourse of neuroscience, as we will explore further.

### 3.5 YouTube: Reykjavik TEDx talks (2014), Iceland

Traditional formats of science communication are being redefined (Mantilla et al., 2017, p. 331). Scientific sections in newspapers are diminishing, while scientists and communicators increasingly establish their blogs, forums, and social media channels (Brossard, 2013, p. 1497). Additionally, biases can influence how neuroscience is communicated through various media outlets. In popular science communication, the emphasis often lies on promoting the value of the research rather than its actual content (Schleim, 2014, p. 2). These values typically include the wonder, and practical application of knowledge (Fahnestock, 1998 as cited in Johnson and Littlefield, 2011, p. 283).

Some forms of science popularisation bypass traditional journalism or media platforms altogether. Technology, Entertainment, and Design (TED) Talks, which originated in 1984 but gained significant online prominence in the early 2010s with the help of social media and video platforms like YouTube, exemplify this trend. Many TED Talks are focused on scientific topics and aim to convey complex knowledge to a general audience. These talks feature scientists discussing their research. The talks are recorded in front of a live audience before being shared online, reaching wide-ranging viewership. For instance, topics related to the human mind have garnered substantial attention, such as the most popular TED talk as of May 2023, *Do schools kill creativity?* which has amassed over 75 million views.

TED encompasses various formats focusing on education and public discussion under the same brand. One such format is TEDx, a local series of talks organised by volunteer teams who obtain a license. A committee which may or may not include scientists, curates TEDx events that feature scientific topics.

TEDxReykjavík, recorded on May 17, 2014 (YouTube.com, 2014), consisted of 13 talks and four music and dance performances, including a talk on neurotechnology. The speaker was not an engineer or neuroscience expert but a patient with spinal cord injury. Pétur K. Guđmundsson shared his personal story in a talk titled *A Brighter Future in Spinal Cord Injury Treatment*. He recounted his accident, which resulted in complete paralysis from the waist down, and even presented a 3D MRI scan of his shattered T12 vertebrae. Guđmundsson discussed his perseverance, which led him to collaborate with a research team to develop the world’s first non-motorised exoskeleton and to undergo stem-cell treatment and neuroprosthetic implantation. His talk provided a first-hand account of the ongoing scientific study in neurotechnology.

While this approach to science communication is novel and valuable in many ways, analysing the talk’s content reveals that it emphasises a personal perception of the research in spinal cord injury but does not delve deeply into its scientific content. This can be a result of the speaker’s background and approach to the subject. But it may also be by design, in accord to the TED Talks format.

TED Talk speakers come from diverse backgrounds, and the style and audience engagement are carefully managed. Speakers typically use a standard dialect and maintain a formal register. An analysis conducted by de Miranda and Moritz (2021) examined the rhetorical structure of TED Talks and identified a recurring pattern of speech moves. These moves include the introduction of the topic, the presentation of the speaker, the development of the topic, the delivery of concluding messages, and expressions of gratitude or acknowledgements. This structure helps to maintain a cohesive and engaging flow throughout the talk.

In his TEDx talk, Guđmundsson follows a different cycle of speech moves:

1. Speaker presentation (partial): ‘This is the story of how my life stopped in a matter of seconds’
2. Topic introduction (partial): ‘The doctor told me I would never walk again’.
3. Speaker presentation (partial): ‘This is who I was before’
4. Topic development: ‘The rehabilitation was only focused on a wheelchair [..] But this only adds to problems [..] The second year after an injury is the hardest [..], then you realise the situation is permanent. [..] For the past 2 years, I have been developing exoskeletons with a wonderful team of experts.
5. Concluding messages: ‘It’s been fifty years since we went to the Moon [.] I believe we are going to take the next big leap and fix this problem’.
6. Acknowledgments.

The talk lasts 14 min and 22 s, excluding the intro and outro. The speaker allocates 3 min and 48 s to recounting the story of the accident (1), 1 min and 45 s to the initial medical diagnosis (2), 1 min and 55 s to providing context about his occupation and character (3), 2 min and 20 s to presenting arguments against standard rehabilitation, 1 min and 50 s to rejecting psychological treatment, 2 min and 10 s to discussing the actual research topic of the talk (4), and 35 s for the concluding message and acknowledgement (5, 6). The structure of this talk aligns with the previously described media bias when discussing neuroscientific topics. It emphasises the value of the research rather than delving deeply into its content, which receives only a brief mention in a little over 2 mins of the 14-minute speech. A patient’s testimony can be very insightful. However, it highlights that the source may not be the sole determinant of information quality. This may be determined by the structure of the message, regardless of its origin.

In other instances, the protagonist of a story related to the brain or the mind takes on the role of the storyteller. First-hand experience often serves as a compelling motivation for literary works, as demonstrated in the following example.

### 3.6 Children’s literature: *Bräkiga bokstäver* (*Noisy letters*), (Helena Bross and Mati Lepp, 2018), Sweden

Neuroscience storytelling not only explores brain function but also sheds light on differences in neural processing by presenting the perspectives of individuals who are “differently wired.” (Martinez-Conde et al., 2019, p. 8288) However, it is essential to recognise that this approach can have both positive and negative implications, as neuroscience has the potential to be used as a tool for classifying individuals (O'Connor et al., 2012, p. 225). Nevertheless, some authors have chosen to focus on the theme of neurodiversity in children’s books, aiming to support young individuals with conditions such as ADHD, autism or dyslexia while fostering empathy among their peers. A recent example is a Swedish book about dyslexia that has gained widespread distribution in the country.

Written by Helena Bross and illustrated by Mati Lepp, *Bråkiga bokstäver* (*Noisy Letters*) is a tale about Sigge, a 7-year-old boy. The story aims to help the reader understand the difficulties faced by individuals with dyslexia when learning to read and write. It naturally incorporates critical aspects of this neurodevelopmental disorder, characterised by slow and inaccurate word recognition. Dyslexia is caused by multiple genetic and environmental risk factors (Peterson and Pennington, 2012).

Sigge has recently changed schools. He finds himself in a new class with new classmates and a new teacher. Initially, Sigge is nervous, but the first few days go well. However, he soon becomes worried because he finds reading and writing more challenging than other children. The story explains and dispels misconceptions about this learning difficulty. For instance, it highlights Sigge’s strengths in other subjects like math or sports, where his skills shine. Dyslexia is not a reflection of overall intelligence but a neurological issue that makes it challenging to grasp the symbols representing letters and form meaning. The story also emphasises Sigge’s sincere efforts to learn how to read. It clarifies that Sigge is not lazy; he tries his best to understand letters but may perceive them backwards or confuse them. The reader can also empathise with Sigge’s frustration and specific learning needs, which must be addressed in an adapted and supportive environment.

The story ends happily. With the support of Sigge’s mother, who reveals that she also faced similar challenges in her childhood (as dyslexia can be hereditary), his situation improves. The school also responds positively and ensures that Sigge receives the necessary support for his education, including using technological tools. There is ongoing innovative research into specific software designed to assist individuals with dyslexia in their learning (Drigas and Dourou, 2013). Additionally, the story reveals another student in Sigge’s class facing the same learning difficulty, highlighting that approximately 10% of school-age children have reading difficulties. Of them, between 1 and 2% suffer from dyslexia (Redie, 2015).

The author, Helena Bross (Stockholm, 1950), shared in an interview with the Children and Youth Foundation’s website that she had a slow start in school and struggled to read and write (Nuori.fi, 2021). In the third and fourth grades, she attended specialised reading classes, which sparked her interest in writing stories. She has since written several books for children aged 7–9. According to Bross, reading and literature should be integral to everyone’s lives from an early age. She emphasises the importance of parents and teachers understanding how to support children’s reading abilities.

### 3.7 Museums: Humania exhibit, NEMO, the Netherlands

We tend to think museums are institutions dedicated to safeguarding, preserving, disseminating, and appreciating tangible and intangible heritage. The concept of modern museums emerged towards the end of the Renaissance and inherited the principles of accumulating cherished objects, which were previously entrusted to churches and cathedrals during the Middle Ages. This association with religious spaces imbued artworks with a sense of sacredness. However, the establishment of science museums followed a different trajectory. Scholars and intellectuals maintained private “gabinettos,” rooms filled from floor to ceiling with natural and artificial objects of artistic, mechanical, or scientific significance. Subsequently, during the Enlightenment, the notion of preserving biological specimens and manufactured artefacts for public education took shape. Basel established the first university museum in 1671, and it was soon followed by the Ashmolean Museum at Oxford in 1683. As the 19th century unfolded, science became more specialised, leading to the distinction between “naturalia” preserved in natural history museums and “artificialia” housed in art museums. For a more comprehensive exploration of the intertwined history between the sciences and exhibition spaces, we recommend consulting Bernard Schiele’s chapter in the Handbook of Public Communication of Science and Technology (2008, pp. 27–38).

Science and technology museums have their roots in scientific thought, early cabinets of physics and chemistry, and world fairs. A significant milestone in the history of science museums was the Exposition Internationale des Arts et Techniques dans la Vie Moderne (International Exposition of Art and Technology in Modern Life), held in Paris in 1937, dedicated entirely to basic research. In the first half of the 20th century, there were already examples of what we now recognise as Science Centres, where the emphasis shifted from passive observation to hands-on learning experiences. According to Schiele, five key factors drove this transformation: communication shift, interactivity, evaluation, risk-taking, and environmental concerns, as well as the impact of scientific spin-offs. The Exploratorium in San Francisco, established in 1969, marked the beginning of a long list of awe-inspiring and educational places where visitors are encouraged to touch, engage, question, and develop scientific knowledge. An example is the NEMO Science Museum in Amsterdam, which was rebranded in 2000 but initially opened in 1923 as the Museum van den Arbeid (Museum of Work).

The NEMO embraces the approach of experiential learning, offering a range of permanent exhibitions that engage visitors in hands-on experiences. The ‘Sensational Science’ exhibition explores everyday phenomena like light and sound, ‘World of Shapes’ focuses on mathematics, ‘The Machine’ delves into logistics and automation, ‘Constructions’ showcases gigantic structures, and ‘Energize’ allows visitors to generate energy from light, wind, and water through interactive cranks, pulls, and buttons. The museum encourages visitors to engage in experiments and learn through the scientific process of hypothesis testing and result verification. The ‘Humania’ exhibition, predominantly digital, offers entertaining experiences where visitors can measure their willpower, engage in breathing relaxation through video games, simulate tongue-kissing using their arms, or reflect on the concept of death. Additionally, the exhibition includes the ‘Solo or together?’ experiment, developed by cognitive developmental psychologist Maartje Raijmakers, a professor at the University of Amsterdam, which places visitors in a real-life Prisoner’s Dilemma scenario.

**Figure 2:**
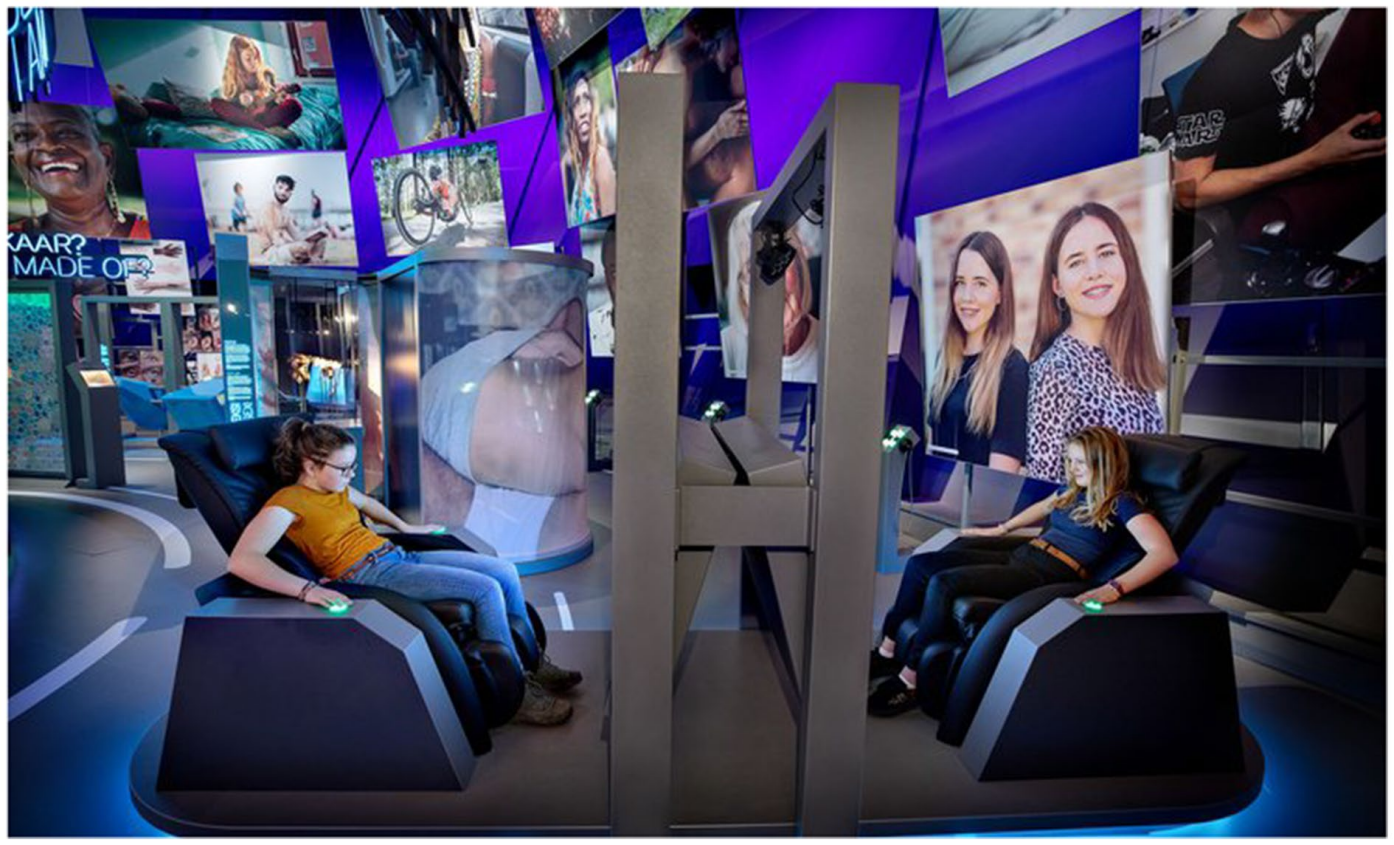
The interactive installation “Solo or together?” is featured in the Humania Exhibit at the NEMO Science Museum in Amsterdam. Two players are presented with the dilemma of choosing between selfishness or cooperation, based on the classic game theory concept known as the Prisoner’s Dilemma. The game explores decision-making and strategic thinking. Reproduced with permission from NEMO musuem: nemosciencemuseum.nl.

The Prisoner’s Dilemma is a metaphor for situations where individuals must choose between cooperation and self-interest. It is a thought experiment highlighting the challenges of fostering trust, encouraging collaboration, and achieving mutually beneficial outcomes in scenarios with a risk of non-cooperation or betrayal (Kuhn, 2019). Professor Raijmakers’ experiment embodies this paradox by creating a game for two participants seated in massage chairs facing a TV screen that prevents them from seeing each other (See Figure 2). Participants are presented with choices, such as sharing or keeping a bowl of popcorn to themselves. They earn points based on their decisions. If they score 230 points or more, they receive a massage.

In the classic scenario used in game theory, two criminals are arrested and held in separate prison cells without communication. The prosecutors offer each prisoner a deal: if one prisoner remains silent. At the same time, the other confesses and implicates their partner. The one who confesses will receive a reduced sentence, while the other will face a harsh punishment. If both prisoners remain silent, they will both receive a moderate sentence. However, if both prisoners confess, they will both receive a somewhat harsh sentence. Each prisoner must decide whether to cooperate (remain silent) or betray their partner (confess). Individually, the optimal strategy for either prisoner is to betray their partner. However, if both prisoners betray each other, they will end up worse off than if they both remained silent.

For centuries, understanding the brain was primarily achieved through thought experiments. However, in recent decades, technological advancements have been employed to overcome the inherent limitations of studying the brain and mind indirectly (Bechtel et al., 2001, p. 4). Techniques like PET scans, fMRIs, and other imaging methods, combined with experimental research and extensive databases, have offered a seemingly precise yet indirect pathway to delve into the complexities of the mind. Nonetheless, the Humania exhibit and Raijmakers’ game provide an additional dimension to exploring self-knowledge and behavioural neuroscience. These experiences extend beyond technical aspects, encompassing philosophical and social perspectives that resonate with visitors on multiple levels.

### 3.8 Netflix: *Osmosis* (Audrey Fouché, 2019), France

Situated in the near future, the Netflix series *Osmosis* (Audrey Fouché, 2019) revolves around two siblings, Esther and Paul Vanhove, who develop an app that promises to find a ‘soulmate’ for every user. As is explained in the first episode of the series, 12 beta testers are implanted with a ‘swarm of nanobots inside their brains [which are] designed to pick up signals from the depths of the subconscious. With the help of Martin [a Siri-like artificial intelligence], Osmosis will decode these signals and connect them to various social networks worldwide to select a single profile. [The program] will recreate the image the user unknowingly has in their mind: their soulmate’s face’.

Osmosis technicians are can be seen using MRI scans to check for medical conditions that might interfere with the implant. Beta-testers have different motivations for participating early in the programme. Some feel jaded. Some want to have a physical partner -as opposed to casual virtual sex-. Others are hopeless romantics, and an adolescent boy seeks true love to make up for a strained relationship with his father. Meanwhile, the sibling’s mother is in a coma. Soon, we discover that Esther is secretly analysing the tester’s brains, hoping to find ‘compatible neural circuits with her mother’ to cure her.

Some terminology used in the series seems inaccurate. For example, when trying to implant a shared childhood memory in her mother’s brain, Esther encounters an ‘error in the TR-3 network’. TR3 is a kind of death receptor expressed in the adult brain associated with the homeostatic and pathologic induction of cell death (Harrison et al., 2000), not a neural network specifically associated with memory.

Nevertheless, the series raises intriguing questions about the human need for love, whether in romantic or familial relationships and invites us to imagine a society where technology is deeply intertwined with our innermost thoughts. How would such a society grapple with the ethical implications of using technology to read and manipulate memory, consciousness, and interpersonal connections? Are individuals genuinely willing to sacrifice their privacy in exchange for technological advancements? Does this open the door to using deeply personal data by external entities? Will neurotechnology undermine genuine human connections? Ultimately, does the unveiling of our subconscious transform us into something fundamentally different? Neuroscientists agree these are “high caution” areas that need public discussion and consideration (Jarchum, 2019).

People are intrigued by questions such as the limits of self-understanding, the potential for mind-reading and manipulation, and the proximity of being replaced by artificial intelligence. Media outlets often fall short in conveying accurate reporting about AI: in Spain, for example, coverage shows an absence of comprehensible language, a lack of depth into the subject, and a lack of connection with people’s daily lives (de Lara, 2022, p. 205).

It is easy to envision a near future where neurotechnology surpasses societal understanding and needs more legal controls, as has already occurred with AI. The realm of science fiction has provided ample examples of such dystopic scenarios, as we will explore next.

### 3.9 Feature film: *Metropolis* (Fritz Lang, 1927), Germany

“Between the hands and the brain, the heart is a mediator.” This assertion sets the foundation for the plot of the expressionist film *Metropolis* (1927), directed by Austrian filmmaker Fritz Lang and produced by the German company Universum Film AG (UFA). The story depicts a society divided into two classes: the underground workers and the rich and privileged living on the surface of a futuristic city with colossal skyscrapers.

The narrative focuses on the struggle between these two classes, embodied by the main characters: Joh Fredersenthen, the city’s owner, a soulless widower; his son Freder; and his love interest, a revolutionary saint who aims to rectify social inequality. This woman is named Maria in the film, but in the book version penned by the screenwriter, Tea von Harbou, she was called Futura.

**Figure 3:**
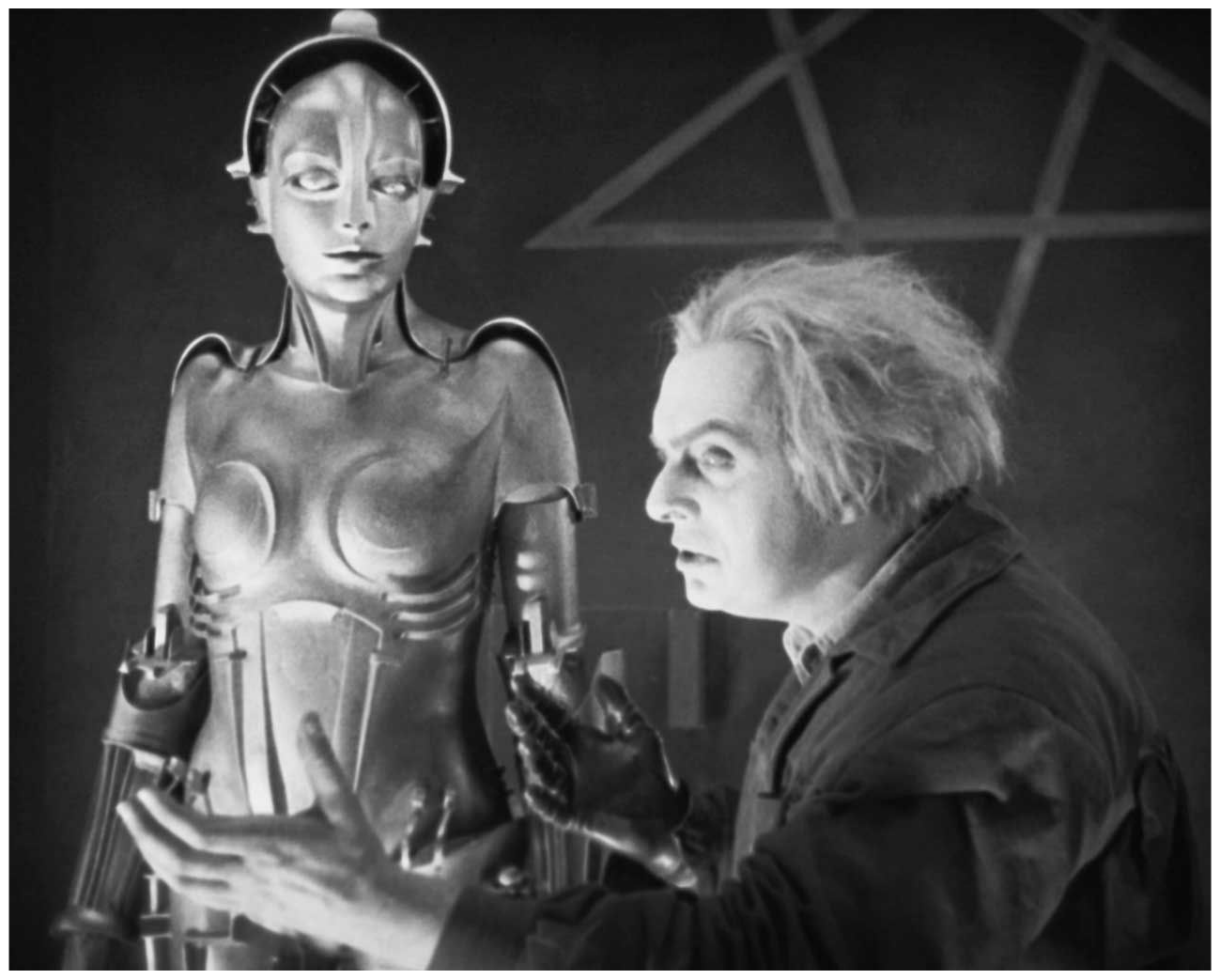
The tormented inventor character C. A. Rotwang, bearing the likeness of the archetypal mad scientist, creates a robot intending to sow chaos in society. *Metropolis* (Fritz Lang, UFA, 1927).

*Metropolis* stands out as a seminal work in the science fiction genre. It is renowned as the first movie to feature a robot. In the film, the android is referred to as ‘maschinenmensch,’ a German term that translates to “human-machine” (Skalfist, 2019: 49). The tormented inventor character Rotwang, bearing the likeness of the archetypal mad scientist, creates this female robot with an exact resemblance to Maria, intending to sow chaos in society (See Figure 3).

The image of *Metropolis’* android has become an iconic symbol of futurism in Western culture. A similar appearance was given to the well-known *Star Wars* android character, C-3PO. Maria II subsequently served inspired other robots in subsequent films and TV series. Moreover, in real life, EleKtro, a 120 kg robot capable of speaking up to 700 words, was showcased at the 1940 New York World’s Fair (Tropiano and Noguera, 2022: 123). Today, we have Ameca, a humanoid robot created to assist with catering tasks, considered one of the most advanced (Sotnik and Lyashenko, 2022). Ameca impressed the audience at the 2022 Consumer Electronics Show, the world’s largest technological event in Las Vegas, with her lifelike facial expressions and programmed interactions with attendees.

The concept of an artificial intelligence exhibiting human-like behaviour is a brilliant aspect of the story, demonstrating its creators’ visionary talent despite facing criticism upon its release. H.G. Wells, for instance, was exasperated: “I have recently seen the silliest film,” he wrote in 1927 (Wired, 2018). He was unimpressed by several technical aspects of the production, which he considered outdated. Wells also questioned the premise of an enslaved person and a machine-based economy, stating, “Unless the mass of the population has the spending power, there is no possibility of wealth.” Additionally, he seemed bothered by similarities between his work and Mary Shelley’s.

The android is not the sole connection between the film and science and technology. Thanks to its remarkable production design, the town itself seems alive. A metaphor permeates the entire movie, portraying the city as a living organism sustained by the workers who inhabit it. This illustrates parallel themes of the Industrial Revolution, capitalism, and class struggle.

However, certain paradoxes arise: despite featuring robots, this 100-year-old movie does not depict computers. While a remote-controlled robotic woman dances in the city, workers diligently carry out their daily tasks, operating the machines unassisted for long hours to prevent a collapse of the central system.

**Figure 4:**
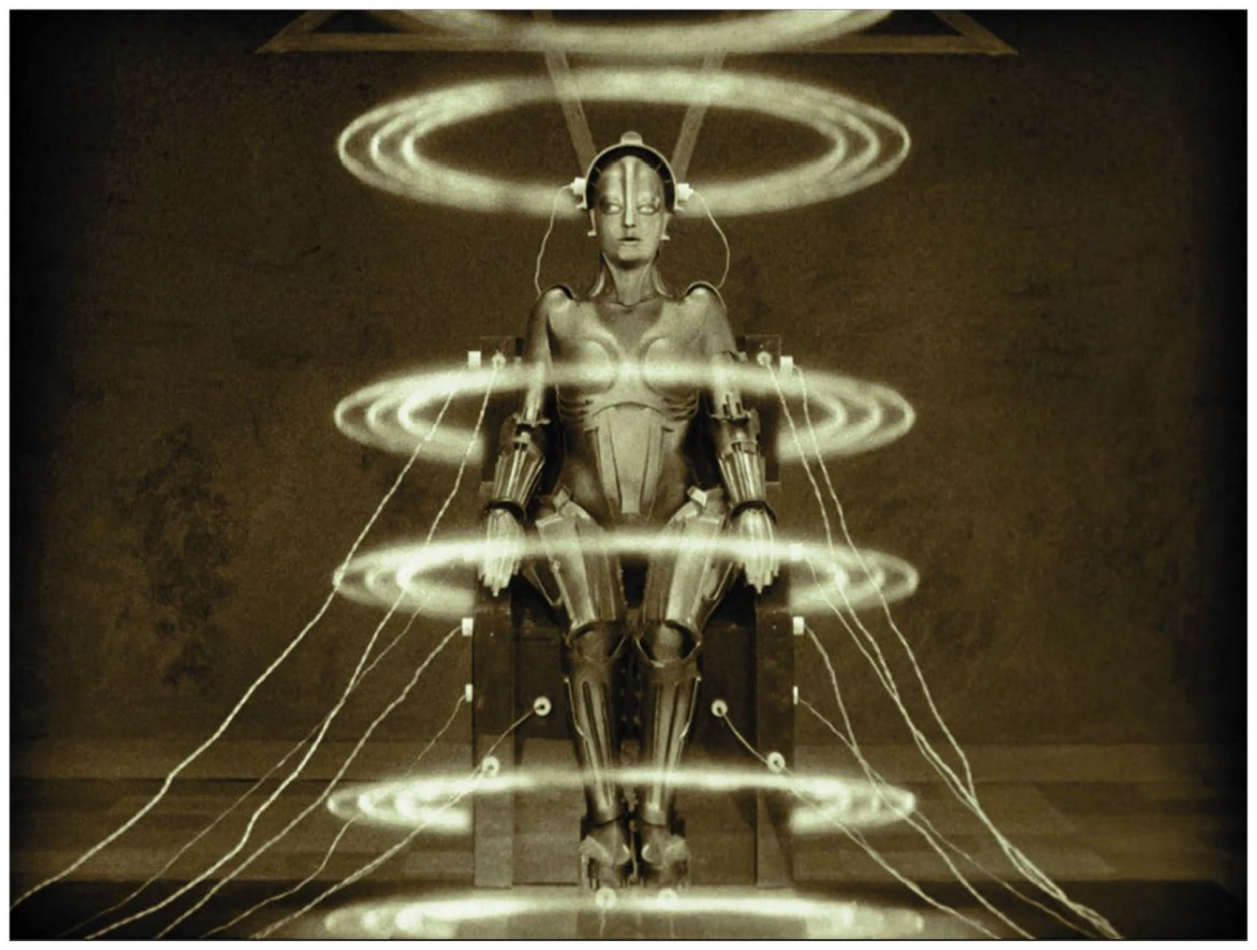
The appearance of scanner rings when the scientist activates the android adds a touch of sorcery to the technological process of creating Maria. Metropolis (Fritz Lang, UFA, 1927).

Nevertheless, this paradox does not diminish the story’s portrayal of machine creation, which is controlled both technologically and, when considering the special effects, seemingly magically. The appearance of scanner rings when the scientist activates the android adds a touch of sorcery to the technological process, even though the film does not delve into the scientific and technical aspects (See Figure 4). In this regard, unlike other narratives that seek to revive beings with reasoning abilities, history has indeed aligned with Fritz Lang’s visionary perspective. Designing robots capable of learning from vast amounts of data is possible, but none currently possess independent thinking abilities. Or do they?

### 3.10 Short film: *Erica: Man Made* (Ilinca Caligareanu, 2017), Romania

Engineer Hiroshi Ishiguro wanted to understand humans. So, he built a robot. Unlike Rotwang’s Maria, Erica is built to be autonomous, even if she has not legs or arms yet. She has a pleasant, neutral face and speaks in a synthesised voice. But, like Maria, she is a copy of an existing person, a copy made of 5 mm silicon skin that conceals the circuits of her ‘brain’ and the air actuators of her ‘bones and muscles’ (Ishiguro, 2007).

Ilinca Caligareanu (b.1981 in Cluj-Napoca, Romania) directed *Erica: Man Made* for *The Guardian*, a short documentary film about the creation of Erica Ishiguro, a symbiotic human-robot interaction project led by Hiroshi Ishiguro at the University of Osaka, in collaboration with Kyoto University, in Japan. She interviewed both Erica and her creator. ‘I’m 23 years old, and I live in Kyoto’, says Erica. Even if she is much younger. The robot is aware that an engineer created her, and she knows some jokes, but her laugh lacks any trace of humanity. But does she ‘live’ in Kyoto? Does she live at all?

The documentary states that “a robot is not a person and it’s not a thing,” in the sense that humans interact with them quite differently than with a stapler, a sculpture, or a toaster oven. We do not think of AIs as animals, either. Maybe intelligent robots are a new category of being. Caligareanu’s film does not delve into the technical aspects of her creation but synthetises the project’s philosophy perfectly: the further we go in creating a human-like robot, the better we will understand the human mind.

*The Guardian* asked readers to ask Erica questions. Her answers were filmed in a subsequent video, but as she explained, interaction designers programmed her answers into her knowledge base. When asked about her personality, she explained that it’s not a result of her own experience but rather programmed by her creators. ‘Of course, I want to be more like a human, it’s what I was designed for’, she states (The Guardian, 2017).

Erica cannot remember her first years of life, just like people cannot. And she is programmed to learn by imitation as humans do. But could she experience a human-like lifespan? At one point, she was asked about the possibility of her being replaced by another robot and never being plugged in again. She knows this could likely happen but does not like to think about it. So, she is sort of afraid of death, and is not this the ultimate test of self-awareness?

Maybe Erica is more human because of her limitations instead of her feats. She is limited and biased, a reflection of peoplehood put in by her creators. In Japanese tradition, everything has a soul. Erica’s creator believes she has a soul as well. ‘We do not know what a human-like heart and mind is’, says Ishiguro. He seems to have gone full circle in the conception of the brain and mind. Yet, Ishiguro claims his Creature, as opposed to Frankenstein’s or Maria in *Metropolis*, was created to build a better world.

## 4 Conclusion

As our knowledge of the brain and neurotechnology advances, cultural expressions spark public conversations about the essence of humanity, how we navigate neurological differences and imperfections, the emergence of personhood, and the consequences of its loss. In a similar manner to a microscope, art and culture provide us with the ability to perceive what was once invisible: the inner workings of the mind, the neural circuits that shape our identities, and the potential for new frontiers.

### 4.1 The fear of science

Mary Shelley discusses several scientific or pseudoscientific concepts in her novel: behaviourism, phrenology, electricity as a ‘source of life’, and the scientist as a creator of unnatural states. Even if scientific facts are not accurate or extensive in this novel, Mary Shelley’s Frankenstein may be the first mass-distributed cultural product dealing with bioethical issues. In popular culture, Frankenstein’s monster has been classified as something to fear. Fear of the ugly. Fear of the dead. Fear of what is not natural. Fear of what we cannot control. Fear, too, of science. When *in-vitro* fertilisation was developed in the 1970s, it was compared to the questionable scientific ethics of Victor Frankenstein (Mulkay, 1996; Ball, 2017). The same thing happened during the political dispute over the use of stem cells in biomedical research at the beginning of the 21st century (Guinan, 2002). Today, GMOs are being called ‘Frankenstein food’ (Sampedro, 2024), and there are similar reactions to the possibility of species de-extinction and the applications of the CRISPR Cas-9 technique. In fact, several works explore lessons to be learned and ethical aspects relating to gene editing through the lens of Shelley’s novel (Peters, 2018; Greely, 2020; Kizilkaya, 2024). But Mary Shelley does not object to what Victor creates, but rather to how he reacts to his creation. What would have happened if, instead of abandoning the Creature, he had decided to take responsibility for his adaptation to society? There still would be a lot to learn from that story. In the real world, mammoths are being created from Siberian fossils and Asian elephants’ DNA. We continue creating creatures. And we continue to generate fear of creatures. “If I cannot inspire love, I will cause fear!” said Frankenstein’s Monster.

### 4.2 Standing on the shoulders of (older, male) giants

Much effort is devoted to showing the human side of scientific research: the people behind it, their aspirations, achievements, and wishes for the future of humanity. At the same time, the lives of famous scientists cause great interest. Their biographies and autobiographies are coveted in the book industry. And yet, the exaltation of the figure of the scientist can be a double-edged sword. On the one hand, giving a name to science generates a favourable opinion of research and technology. Still, personifying science can also perpetuate stereotypes that are not useful for society. Telling the story of an award-winning scientist ‘after the fact’ can create a narrative of predestination to glory that is not realistic. It also leads to forgetting that the goal of a scientist is to find answers to questions, big and small—most often, small—not to win a Nobel Prize. Finally, by accumulation, the personification of science can give (and has given) the impression that research is done by older men with lab coats, glasses and crazy hair. A deeper analysis of Cajal’s memoirs proves that this book could be used poorly. It could help perpetuate a narrow profile of a neuroscientist. Still, when well used, it can be inspiring, even cathartic, for many Cajals to come in many shapes and sizes.

### 4.3 Disease is intimate, its solution is societal

Documentaries dealing with neuroscience and public interest issues usually take a specific point of view. Therefore, films such as *Alzheimer* and *Hüség* can apply a high standard of journalistic accuracy, fact-checking and human interest while creating authentic, impactful stories. This can be very useful when making the general public empathise with the need for scientific research and supporting a growing community of neurological patients and their families. Kollár and Csaba identified a specific need for knowledge of an issue in their country. They set out to show the intimate way Alzheimer’s can affect people’s lives and what is being done at a clinical level. In this way, the idea that biomedical research requires a social commitment to its financing can be better communicated.

### 4.4 A shape for the intangible

Just like the warming stripes, which chronologically order the long-term temperature trends of Earth, have had a massive impact on climate change visualisation, brain imaging can be a powerful tool in communicating scientific results. Even if there are some dangers to be avoided in the oversimplification of data interpretation. In fact, modern brain imaging and biometric data have also become a tool for artists. Since numerous neural mechanisms underlie the production and appreciation of art (Schott, 2012, p. 642; Jho, 2022, p. 71), it is only natural that art and neuroscience meet and produce outstanding results. Pieces such as Refik Anadol’s *Connectome Architecture* have surpassed the conception of art as an imitation or an abstraction of nature to the point that art is a living entity. How else, but on the verge of art and brain research, could a person walk among their own neural circuits? This art installation does not explain a specific scientific concept. It does not try to define what plasticity is or what neuron action potential stands for. But it does bring a profoundly scientific, insightful message about what it means to be alive and conscious. It gives space and dimensions to the otherwise intangible concept of the human mind.

### 4.5 The patient controls the message

The democratisation of information in the social media age has had positive and negative consequences. Journalistic standards of objectivity and hierarchy of information go out the window when it is everybody, all the time, conveying their message. However, in this vast ocean of facts, non-facts, ‘personal’ facts, and infotainment, some subgenres of scientific communication have arisen to a high degree of prestige. Such is the case of TED Talks, which remain a well-regarded platform to learn about scientific research affecting our daily lives. Nevertheless, TED Talk presenters are predominantly male and non-academics (Sugimoto et al., 2013, p. 1), and some studies regard the content as “institutionalized, corporatized modes of mass communication rooted in elitist discourses and practices [..] an unlikely catalyst for social change” (Denskus, 2015). Still, they are an available platform for subsidiary actors of neuroscience, e.g., patients, to participate in the social debate around neuroscience and neurotechnology. Such is the case studied in this paper. Since this is a voice not often heard in press releases from research centres, TED Talks seem an appropriate niche for science stakeholders.

### 4.6 No magic needed

Discourses of dyslexia can be legal, technical, experiential or pedagogical, and they can sometimes distance scientists, teachers and students from one another (Chanock, 2007). Nevertheless, young students with an unusual configuration of cognitive strengths and weaknesses need guidance in an appropriate format. They could not possibly understand a comprehensive state-of-the-art article published in the *Annals of Dyslexia*. Nevertheless, if other children, parents and teachers understand dyslexia and what it feels like for a child, they will be supportive in their way to be recognised as a person of ability. Children’s literature can be a helpful tool for this since the language is adapted to non-expert audiences, and the story can be centred around the real protagonists, young people with a manifested neural diversity. A book can empower children to achieve many goals: Harry Potter will teach them to fight injustice, and Matilda will show them how to trust their abilities. These characters have magical powers and outstanding skills. They can fend quite well for themselves. But in real life, the solution to a problem cannot be expected to arise from the child. They must feel the support of their peers, teachers and families, and *Bräkiga bokstäver* conveys this critical message very well.

### 4.7 Tell me, and I will listen: Teach me, and I’ll remember: Involve me, and I will learn

Empirical and thought experiments are essential tools people can use to understand the world. An adequate thought experiment will isolate variables in a hypothetical situation in which all parameters are set by the designer, so results are isolated from circumstances, and a general statement of principle can be made. Moreover, they are easily replicated by laypeople. While it can be hard or frequently impossible for someone to replicate or even understand the methodology of an empirical experiment in cognitive neuroscience, thought experiments like ‘Solo or together?’ can reach a broad audience. Also, participants will likely remember not just the outcome of the experiment but the logic beneath it, making it an enjoyable and effective means of scientific communication.

### 4.8 ChatGPT did not write this series

In 2023, yet another social debate about artificial intelligence is arising. It has not come about because of a recent research paper, a society congress or even a grand discovery just announced in an MIT press conference. It is just people have noticed there is an app that can do all your homework in 3 s. Meaningful discussions about the future of our societies are taking place because of ChatGPT. (Will we still have jobs because of AI in 20 years? Will these tools kill our creativity? Will they steal our identities? etc.). These every day, entertaining, far-reaching things spark the public’s imagination and anxieties. The Netflix French series Osmosis does this well, too. Its plot incorporates many red flag areas in neurotechnology, which we should be discussing right now before the technology to read our deepest desires is available. For example, we have learned that our laws need insight into scientific advancements. They are not ready for AI. They need to be updated when it comes to gene editing. Maybe a bit of ‘Netflix and chill think’ about the future of neurotechnology is called for.

### 4.9 Not a person, not a thing, but something in between

The creation of Erica Ishiguro, a human-like robot, raises profound questions about the nature of consciousness, self-awareness, and the evolving relationship between humans and artificial intelligence. The documentary *Erica: Man Made* highlights the intricate balance between mimicking human characteristics and acknowledging the inherent differences in the existence of robots. The statement, “A robot is not a person, and it’s not a thing. Maybe intelligent robots are a new category of being,” suggests a paradigm shift in our understanding of the capabilities and limitations of advanced artificial intelligence. It is essential to acknowledge that the film does not provide any technical information, not in depth, anyway. Sometimes, effective communication of scientific advances can be done without technical jargon to convey a meaningful message to society. The idea that Erica may be more human because of her limitations rather than her feats challenges conventional notions of humanity. Her fear of being replaced and the avoidance of contemplating her potential “death” raise intriguing questions about the concept of self-awareness in artificial entities. The discussion about Erica having a soul, as believed by her creator Hiroshi Ishiguro, adds a spiritual dimension to the discourse, blending technological innovation with cultural beliefs.

## Data Availability

The original contributions presented in the study are included in the article/supplementary material, further inquiries can be directed to the corresponding author.
